# Co-circulation of a novel phlebovirus and Massilia virus in sandflies, Portugal

**DOI:** 10.1186/s12985-015-0407-0

**Published:** 2015-10-24

**Authors:** Fátima Amaro, Líbia Zé-Zé, Maria J. Alves, Jessica Börstler, Joachim Clos, Stephan Lorenzen, Stefanie Christine Becker, Jonas Schmidt-Chanasit, Daniel Cadar

**Affiliations:** Centre for Vectors and Infectious Diseases Research, National Institute of Health Ricardo Jorge, Águas de Moura, Portugal; BioISI-Biosystems and Integrative Sciences Institute, University of Lisboa, Faculty of Sciences, Campo Grande, Lisboa Portugal; Bernhard Nocht Institute for Tropical Medicine, WHO Collaborating Centre for Arbovirus and Haemorrhagic Fever Reference and Research, Hamburg, Germany; Institute for Parasitology, University of Veterinary Medicine Hannover, Hannover, Germany; Institute of Infectology, Friedrich-Loeffler-Institute, Federal Research Institute for Animal Health, Greifswald, Germany; German Centre for Infection Research (DZIF), Hamburg-Lübeck-Borstel, Hamburg, Germany

**Keywords:** Phlebovirus, Alcube virus, Salehabad complex, Complete genome, Phylogeny

## Abstract

**Background:**

In Portugal, entomological surveys to detect phleboviruses in their natural vectors have not been performed so far. Thus, the aims of the present study were to detect, isolate and characterize phleboviruses in sandfly populations of Portugal.

**Findings:**

From May to October 2007–2008, 896 female sandflies were trapped in Arrábida region, located on the southwest coast of Portugal. Phlebovirus RNA was detected by using a pan-phlebovirus RT-PCR in 4 out of 34 *Phlebotomus perniciosus* pools. Direct sequencing of the amplicons showed that 2 samples exhibited 72 % nucleotide identity with Arbia virus, and two showed 96 % nucleotide identity with Massilia virus. The Arbia-like virus (named Alcube virus) was isolated in cell culture and complete genomic sequences of one Alcube and two Massila viruses were determined using next-generation sequencing technology. Phylogenetic analysis demonstrated that Alcube virus clustered with members of the *Salehabad virus* species complex. Within this clade, Alcube virus forms a monophyletic lineage with the Arbia, Salehabad and Adana viruses sharing a common ancestor. Arbia virus has been identified as the most closely related virus with 20-28 % nucleotide and 10-27 % amino acid divergences depending on the analysed segment.

**Conclusions:**

We have provided genetic evidence for the circulation of a novel phlebovirus species named *Alcube virus* in *Ph. perniciosus* and co-circulation of Massilia virus, in Arrábida region, southwest of Portugal. Further epidemiological investigations and surveillance for sandfly-borne phleboviruses in Portugal are needed to elucidate their medical importance.

## Findings

The world-wide impact of sandflies (Diptera: Psychodidae) as significant public health concern and vectors of several zoonotic diseases affecting humans is well known. The most widely distributed pathogens transmitted by sandflies are protozoan parasites of the genus *Leishmania*, but they also act as vectors of several phleboviruses (*Phlebovirus* genus, *Bunyaviridae* family) that are associated with human illness (from transient febrile illness to severe neuroinvasive diseases) [1–3]. Based on antigenic and genomic relationships, six species complexes of phleboviruses are recognized and/or proposed so far for which transmission by sandflies has been shown [4–10]. In the Mediterranean Basin, phleboviruses are emerging agents of infectious diseases whose real medical importance has not yet been fully addressed. Until very recentely only Toscana virus, a member of the *Sandfly fever Naples virus* species complex was known to circulate in Portugal after first being reported in 1985 when a tourist was infected in the Algarve region, in the south of the country [11, 12]. However, no entomological surveys to detect phleboviruses in their natural vectors have been performed so far. The aims of the present study were to detect, isolate and characterize phleboviruses in sandfly populations of Portugal.

**Fig. 1:**
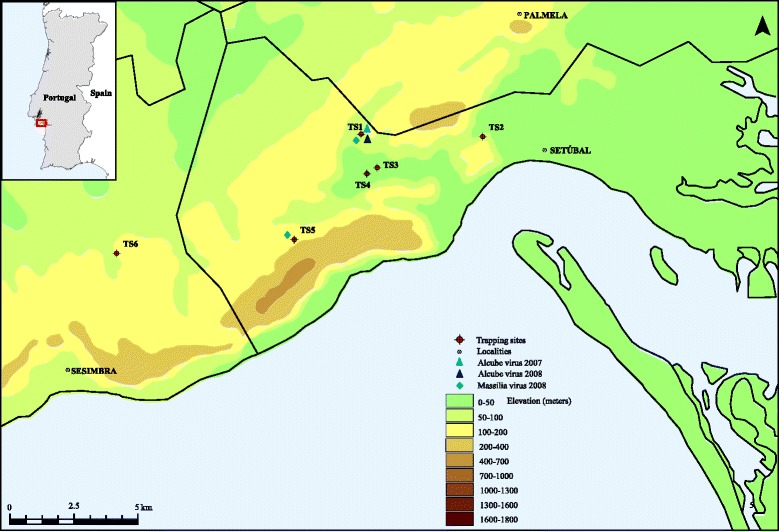
Map depicting the geographic location of the trapping sites from where the sandflies have been collected and the positive sites where the Alcube and Massilia viruses were detected

**Fig. 2:**
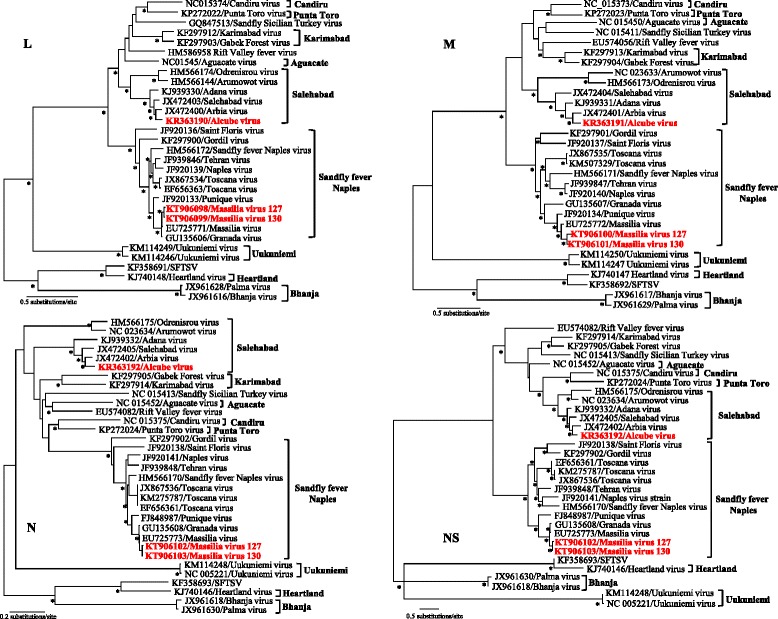
Phylogenetic analyses based on complete genomes of the selected members of the Phlebovirus genus, including the putative novel Alcube virus species and two new Massilia viruses. The phylogenetic trees were inferred on the basis of complete L, M and S (N and NS) protein sequences with the use of the Bayesian Markov chain Monte Carlo method in MrBayes v3.2.5 (http://mrbayes.sourceforge.net/index.php) and, in parallel, the maximum-likelihood methods using PhyML v3.0 (http://www.atgc-montpellier.fr/phyml/). Statistical support of grouping from Bayesian posterior probabilities (clade credibilities ≥90 %) and maximum-likelihood bootstrap replicates (≥70 %) is indicated with an asterisk. The taxon information includes the GenBank accession number and virus abbreviation. The recognized and/or proposed species or species complexes of phleboviruses are indicated to the right of the trees. The Alcube virus and Massilia viruses generated during this study are bolded. The scale bar represents amino acid substitutions per site

In order to address the lack of information about phlebotomine sandflies as vectors of phleboviruses in Portugal, field collections were made in Arrábida region, located on the southwest coast of Portugal (Fig. 1). CDC miniature light traps modified with ultra-fine mesh were placed at dusk and checked after sunrise, for three consecutive nights at monthly intervals. The traps were set near or inside animal housing facilities such as kennels, hen houses, rabbit hutches, horse stables and sheep pens in six different trapping sites (Fig. 1). Collected specimens were aspirated, placed in tubes and immediately frozen at–80° for processing in the laboratory. From May to October 2007–2008, 896 non-engorged female sandflies were trapped, morphologically identified as *Phlebotomus perniciosus* and subsequently grouped in pools of up to 60 specimens. Pools of sandflies were homogenized and suspended in Hank’s solution containing 7.5 % bovine albumin and antibiotics. Total RNA and DNA from homogenized sandfly pools were extracted using RTP® DNA/RNA Virus Mini Kit according to the instructions of the manufacturer (STRATEC Biomedical, Birkenfeld, Germany). Extracted samples were analyzed for the presence of phlebovirus RNA by using a pan-phlebovirus RT-PCR published elsewhere [13]. Of 34 pools processed, we detected phlebovirus RNA in 4 pools of female *Ph. perniciosus* trapped in two distinct trapping sites (Fig. 1). Direct sequencing of the pan-phlebovirus PCR amplicons showed that 2 samples collected from the same trapping site but from different years exhibited 72 % identity with Arbia virus, and two (originated from this and another trapping site) showed 96 % identity with Massilia virus, both members of the *Phlebovirus* genus. Isolation attempts were made for the positive samples. Briefly, 100 μl of the supernatant of the sandfly pool homogenates were used to seed Vero E6 cell monolayer flasks. A successful isolation was achieved, without the need of blind passages, from one Arbia-like virus positive pool sample. The complete genomic sequence of the Arbia-like virus isolate and the two Massilia virus strains was determined using next-generation sequencing technology. The cell-culture supernatant was filtered and nuclease-treated. RNA and DNA from enriched viral particles were extracted, reverse-transcribed, fragmented, ends-repaired, dA-tailed, adaptor-ligated and purified. All library preparations were performed using the NEBNext® Ultra™ DNA Library Prep Kit for Illumina® (New England Biolabs, Inc. USA) and analyzed in an Illumina MiSeq run of 250-bp end reads. Complete genome assembly, sequence analysis, and multiple alignments were performed using Geneious v7.1.8 (Biomatters, Auckland, New Zealand). The new virus is designated Alcube virus since the isolate was obtained from sandflies collected near a small river named Alcube. The complete genome sequence was determined and used to compare the genetic and phylogenetic relationships with other phleboviruses. Alcube virus exhibits the characteristic phleboviruses genome organization with L (encoding the RNA-dependent RNA polymerase), M (encoding the non-structural protein NSm and the two glycoproteins, Gn and Gc), and the S segment (encoding the nucleocapsid protein, and a non-structural protein NSs in an ambisense manner) (GenBank acc. no. KR363190-KR363192). The complete sequence comprises 1,758 nt, 4,164 nt, and 6,405 nt for the S, M, and L segments, respectively. Genetic distances of the Alcube virus were compared with the members of the Salehabad virus antigenic complex and other representative phleboviruses (Table 1). Arbia virus has been identified as the most closely related virus with 20-28 % nucleotide and 10-27 % amino acid divergences depending on the analysed segment (Table 1). Alignment of the deduced amino acid sequences coded by the ORFs of S, M and L segments by using the CLUSTAL plugin in Geneious v7.1.8 and subsequent phylogenetic reconstruction using a maximum-likelihood method (WAG + G model) in PhyML and parallel Bayesian Markov Chain Monte Carlo (MCMC) method implemented in MrBayes 3.0 software (data not shown) demonstrated that Alcube virus clustered with members of the *Salehabad virus* species complex. Within this clade, Alcube virus forms a monophyletic lineage with the Arbia, Salehabad and Adana viruses sharing a common ancestor (Fig. 2). Furthermore, phylogenetic analyses of the L, M, and S segment sequences were consistent with earlier reports [7, 8], confirming that viruses belonging to the Salehabad virus antigenic complex cluster together, and the absence of branching inconsistencies suggests no reassortment events among these viruses. Functional domains and amino acid motifs in the viral proteins that are conserved among other known phleboviruses were also identified in proteins of the Alcube virus (data not shown). In the polyprotein, the signal sequences, transmembrane domains, cleavage sites for the cellular signal peptide peptidase, and Golgi retention signals for Gn and Gc are conserved as observed for the other members of the Salehabad serocomplex [7]. However, Alcube virus exhibits little variation in the patterns of predicted glycosylation sites from those of the Salehabad serocomplex members (data not shown). Massilia virus strain W is most closely related with the two Massilia virus strains from Portugal with 6-19 % nucleotide and 2-14 % amino acid divergences depending on the analysed segment. Phylogenetic reconstruction using complete genomes demonstrated that the two Massila virus strains from Portugal (GenBank acc. no. KT906098- KT906103) clustered with members of the *Sandfly fever Naples virus* species complex (Fig. 2).

**Table 1:** Genetic distances (nucleotide and amino acid divergences) between sequences of the L, M, and S (nucleocapsid, N; nonstructural, NS) genes and proteins of selected phleboviruses and Alcube virus

Nucleotide/amino acid divergences (%)												
Protein\Virus	ARBV	SALV	ADAV	AMTV	ODRV	RVFV	KARV	PTV	MASV	SFNV	TEHV	TOSV
L	23.2/10.6	23.9/10.9	26.4/15.6	38/37.5	38.4/36.5	40.2/41.9	40.6/43.6	43.5/43.1	42.7/46.7	42/45.5	42.5/46.2	42.5/46.2
M	27.9/21.4	37.6/36.9	31.6/27.5	55.1/66.7	53.4/65.6	53.1/ 66.8	53.5/64.7	58.6/66.5	56.7/66.9	56.5/66.8	58.1/67.5	57.3/67.8
N	19.7/9.8	21.3/10.5	27.3/21	44.8/53.7	44.5/50.8	42.7/49	44.4/48.6	42.5/46.3	44.8/52.8	46.2/55.2	47.6/55.2	46.2/54.4
NS	28.2/27.1	33.1/30	32.1/29.9	53.2/62.9	50.9/61.5	69.7/83.1	62.8/75.6	77.1/83.2	70.8/87.6	71.9/86.9	70.9/89.1	70.8/88.3

*ARBV* arbia virus, *SALV* salahabad virus, *ADAV* adana virus, *AMTV* arumowot virus, *ODRV* odrenisrou virus, *RVFV* rift valley fever virus, *KARV* karimabad virus, *PTV* punta toro virus, *MASV* masillia virus, *SFNV* sandfly fever naples virus, *TEHV* tehran virus, *TOSV* toscana virus

To date, Toscana virus [11, 12] from the *Sandfly fever Naples virus* species complex is the only phlebovirus known to circulate in sandfly populations of Portugal. In this study, a novel phlebovirus species, designated *Alcube virus*, was discovered in phlebotomine sandflies (*Ph. perniciosus*) in Arrábida region, located on the southwest coast of Portugal. The virus was isolated and characterized using full-length genome sequence data. Phylogenies demonstrated that Alcube virus clustered with members of the *Salehabad virus* species complex within the genus *Phlebovirus* and forms with the Arbia, Salehabad and Adana viruses a distinct monophyletic lineage within this clade. In Turkey, high Adana virus seroprevalence rates in goats, sheep, and dogs were demonstrated [14]. In contrast, low seroprevalence rates in humans suggest that Adana virus is not likely to constitute an important public health problem in Turkey [14]. In the Arrábida region, we also detected the presence and co-circulation of Massilia virus, a member of the Sandfly fever Naples serocomplex which was described only in France so far. This is the first report of Massilia virus outside France. The medical and public health impact of Massila virus in Portugal remains to be investigated. Although the number of sandflies trapped was relatively small, the number of phlebovirus-infected pools were similar to those reported from France and Italy [15, 16]. These findings suggested that a relatively high proportion of sandflies are naturally infected. The results of this study calls for further epidemiological investigations and surveillance for sandfly-borne phleboviruses to elucidate their medical and veterinary importance.

In conclusion, we have provided genetic evidence for the circulation of a novel phlebovirus species named *Alcube virus* in *Ph. perniciosus* and co-circulation of Massilia virus, a previously recognized phlebotomine-borne phlebovirus, in Arrábida region, southwest of Portugal.
